# Growth of liver transplantation in the United Arab Emirates: lessons from the national deceased organ donation program and international collaboration

**DOI:** 10.3389/ti.2026.16160

**Published:** 2026-10-05

**Authors:** Shiva Kumar, Ali Al Obaidli, Maria Gomez, Mustafa Al Mousawi, Giuseppe Iuppa, Luis Campos

**Affiliations:** 1 Digestive Disease Institute, Cleveland Clinic Abu Dhabi, Abu Dhabi, United Arab Emirates; 2 National Center for Regulating Donation and Transplantation of Human Organs and Tissues, UAE Ministry of Health and Prevention, Dubai, United Arab Emirates; 3 Kuwait Organ Procurement Unit, Kuwait City, Kuwait

**Keywords:** liver transplantation, deceased organ donation, international collaboration, organ sharing, organ utilization

## Introduction

Deceased organ donation in the United Arab Emirates (UAE) was enabled by passage of the landmark federal Law No. 5 in 2016, which formally recognized brain death and subsequent decree from the Ministry of Health and Prevention in 2017 enabling confirmation of brain death, following which transplantation of deceased donor abdominal and thoracic organs was successfully established [1, 2]. In 2023, Johnston-Webber et al. proposed a conceptual framework to evaluate national donation programs across four domains: governance, financing, service provision, and information, supported by enabling factors [3]. After applying this framework to the UAE, two framework extensions were identified: healthcare workforce support and trust as an additional enabler, with international collaboration as a fifth domain, which have remarkably impacted the growth of liver transplantation in the UAE. We summarize the growth and success of deceased organ donation and liver transplantation in the UAE and present an interim status report after the first seven operational years, highlighting the impact of successful international collaboration including regional organ sharing to meet the needs of our patients.

### History of Liver Transplantation in the Middle East and UAE

Historically, apart from the Kingdom of Saudi Arabia (KSA), liver transplantation among the Gulf Cooperation Council (GCC) and Arabic countries relied largely on living donor liver transplantation (LDLT). Various factors have contributed to this, including limited awareness of deceased organ donation, as well as perceived cultural and religious considerations [4]. Moreover, with the lack of clarity on definition of brain death, many countries in the region historically relied on patients traveling abroad to seek transplant services or collaboration with established high-volume liver transplant centers in the West [5–7].

In the UAE, despite the lack of assigned infrastructure for organ donation and transplantation, considerable progress has been made since inception in meeting the needs of patients needing liver transplantation. This was made possible by seamless collaboration between numerous stakeholders, including but not limited to the UAE National Committee for Organ Donation and Transplantation, Ministry of Health and Prevention (MOHAP), Department of Health in Abu Dhabi (DOH), Dubai health authority (DHA), M42 healthcare, Cleveland Clinic Abu Dhabi (CCAD), and PureHealth, among others. One of the most consequential early initiatives was the establishment of an organ procurement organization (OPO) to coordinate organ donation and procurement within the country, with regulatory oversight provided by MOHAP, DOH and DHA. Other key initiatives included strategic workforce planning and recruitment, coupled with efforts to increase awareness among healthcare professionals about deceased organ donation.

Since the first deceased donor LT in February 2018 and LDLT in July 2018, 376 liver transplants have been performed in the UAE until the end of 2024, including 34 LDLTs. Even though CCAD remains the only multi-organ thoracic and abdominal transplant center in the nation, more recently three other liver transplant centers were established - at King’s College Hospital London Dubai in late 2023, followed by Burjeel Medical City and Sheikh Khalifa Medical City in 2024, both in the emirate of Abu Dhabi that had respectively performed 16, 14 and 2 liver transplants by the end of 2024 (Figure 1) Even though patients in the UAE historically travelled abroad for liver transplantation, this is no longer the case since the establishment and growth of liver transplantation.

**FIGURE 1:**
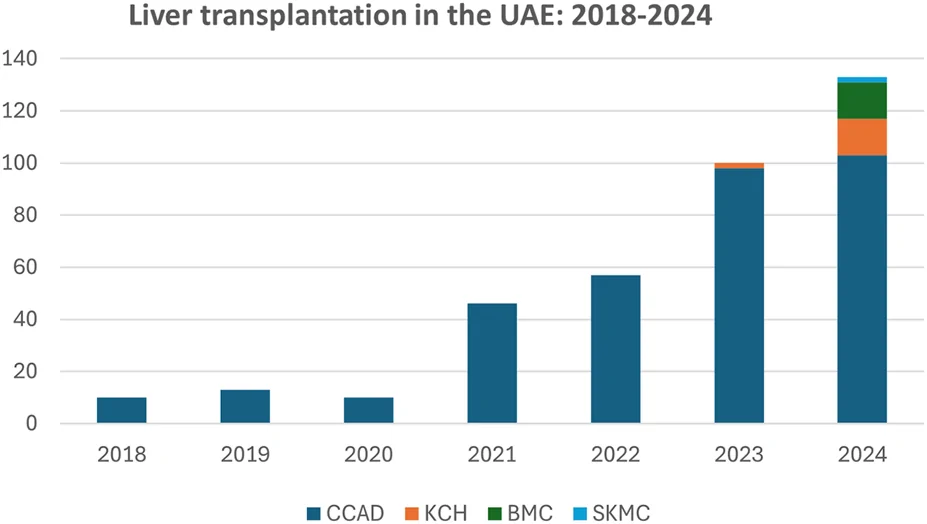
Number of liver transplants performed in the UAE: 2018–2024. CCAD,Cleveland Clinic Abu Dhabi; KCH, Kings College Hospital London, Dubai; BMC, Burjeel Medical City, Abu Dhabi; SKMC, Sheikh Khalifa Medical City, Abu Dhabi.

This report summarizes the current status of liver transplant in the UAE, progress made since inception and our experience to date, highlighting the impact of expanded regional collaboration, while outlining future considerations, challenges and opportunities for continued growth and expansion.

## Methods

### Conceptual framework analysis to expand deceased organ donation in the UAE

Historically, deceased organ donation in the Middle East region has been limited, with only eight countries reporting any activity in deceased donation, with an average donation rate of 5.95 deceased donors per million population (PMP).^1^ The relative lack of established programs in deceased organ donation in the region and limited awareness of the same among both the public and healthcare professionals posed major challenges in ensuring the continued growth in deceased organ donation and solid organ transplantation.

The Johnston-Webber et al. conceptual framework evaluates national donation programs across four domains: Enabling elements analysing the governance and regulatory framework of the system, structural elements including financing and information technology, operational elements related to service provision, and capacity building [3]. We conducted a narrative review of UAE organ donation and transplantation policy, annual reports, and registry data between 2017 and 2024.

### Enabling elements: governance and regulatory framework

Transplantation of human organs from both deceased and living donors in the UAE is regulated by federal Law No. 5 of 2016 which formally recognized brain death, with subsequent clarification of the legal definition of brain death by a ministerial decree in 2017 from the Ministry of Health and Prevention, paving the way for deceased donor organ transplantation in the country*.* ^2^ More recent legislation in 2023 (UAE Federal Decree-Law No. 25), aligned with the objectives of the 77th World Health Assembly resolution, classified organ donation and transplantation as essential healthcare services, thereby mandating their coverage under health insurance with the goal of ensuring equitable access to transplantation services irrespective of gender, ethnicity, religion, social or financial status.^3^ It also aims to promote organ donation, ensure fair distribution of organs, protect the rights of donors and recipients, and combat trafficking in human organs and tissues. The legal framework for deceased organ donation in the UAE relies on explicit consent from next of kin.

While public support and trust are widely recognized as key enablers of deceased organ donation programs, our experience in the UAE highlights that establishing and sustaining the trust and engagement of the healthcare workforce is an equally fundamental prerequisite for implementation and growth of deceased organ donation as they are likely to interface with deceased donor families prior to organ donation decisions [8]. Physicians, nurses, and coordinators are at the forefront of donor identification, family approach, and organ retrieval; thus, their confidence in the national program and its processes is critical. Introducing deceased donation into a healthcare system requires clinicians to adopt new practices, often under complex clinical circumstances. To ensure success, the workforce must perceive the program as credible, transparent, equitable, and aligned with international best practices. This requires structured training, clear national guidelines, and institutional support mechanisms that empower professionals to act with confidence. The need to highlight this dimension independently stems from the fact that building healthcare workforce trust requires specific strategies, focused efforts, and dedicated resources to ensure that professionals are prepared, supported, and motivated to lead the donation process. Building this foundation of workforce trust and capacity enables the consistent delivery of high-quality donation practices and strengthens the credibility of the program in its formative years.

### Structural elements

Another landmark development was the establishment of a National Center for Regulating Donation and Transplantation of Humans Organs and Tissues (NCDT) within MOHAP in September 2020. This center, led by professionals with experience in deceased organ donation, has helped unify and provide critical oversight to national efforts in the field of organ donation and transplantation. The dual role of the NCDT in the early phase, functioning both as a regulatory agency and as the operational arm responsible for managing the UAE OPO, has been instrumental in establishing trust, unifying the system, and providing medicolegal support throughout the deceased organ donation process. This integrated approach has facilitated the coordination and implementation of organ donation protocols across the hospital network, ensuring compliance with national regulations while enhancing the efficiency and reliability of the donation system. NCDT is responsible for oversight of all organ transplant activities in the country, including maintaining registries of organ donors and wait-listed recipients, providing oversight of the deceased organ donation pathway including donor identification and referral across all UAE hospitals, consent, determination of donor eligibility including serologic testing, organ allocation including regional organ sharing collaboration as well as tracking of key performance measures related to deceased organ donation. At present, post-transplant outcomes are reported by individual transplant centers, with regulatory oversight provided by the Ministry of Health and Prevention, Department of health in Abu Dhabi and the Dubai health authority in Dubai. Resource allocation to support deceased donation also entailed the establishment of organ donation and critical care support units within the largest hospitals in the country, staffed by critical care professionals, and entrusted with the early identification and management of potential brain-dead donors while optimizing chances for deceased donation. At present, these units are jointly staffed and resourced by the NCDT and participating hospitals.

The UAE operates under an opt-in framework and, notably, does not yet have donor registration linked to a driver licensing system. Despite the absence of these conventional policy levers, the UAE has achieved rapid growth by prioritizing a health system–centric strategy that integrates organ donation into routine clinical practice. This includes intensive capacity building of healthcare professionals, implementation of standardized national policies and procedures, establishment of a unified coordination model, and strong leadership engagement at institutional and governmental levels. Central to this approach is the development of trust in the system among both healthcare providers and the public, demonstrating that organizational effectiveness and clinical integration can, in certain contexts, compensate for the absence of large-scale registry infrastructure.

In parallel, the authors recognized that public-facing strategies remain essential for long-term sustainability. Initiatives such as donor pledge programs, alongside effective and transparent communication with media and the broader public, are critical to strengthening societal awareness and acceptance of organ donation. Accordingly, the UAE is in the process of implementing these strategies to complement its health system approach and to further sustain and expand the national program.

### Operational elements, capacity building, training and research

With the overarching goal of creating a self-sufficient deceased donation program in the UAE and the region, various educational collaborative partnerships with local and international professional societies and institutes were established, which were the driving force behind intensive educational initiatives for professionals involved in deceased organ donation. Given the diversity in training backgrounds of the healthcare workforce in the UAE, training critical care professionals on the unified global criteria of brain death following the publication of the world brain death project played an important role in enhancing support for and timely conversations with families about deceased organ donation [9]. Collaborative initiatives with respect to best practices in deceased organ donation were established with high performing organ donation programs in the USA and Europe, including implementation of a quality assurance program for organ donation with key performance measures to track performance of organ donation units, and improvements in identification, consenting and optimization of potential brain-dead deceased donors.

### Donor evaluation

Donor evaluation in the United Arab Emirates presents unique challenges driven by the country’s highly diverse and predominantly expatriate population. With approximately 85% of residents originating from multiple geographic regions, including areas endemic for infectious diseases such as tuberculosis, Dengue fever and malaria, comprehensive risk assessment is essential to ensure transplant safety. A key limitation is the difficulty in obtaining complete past medical histories, particularly for recently arrived or transient populations. Consequently, the national program has strengthened donor evaluation protocols through enhanced laboratory screening, standardized clinical assessment pathways, and risk stratification approaches to mitigate infectious and epidemiological uncertainties while maintaining organ utilization. These practices are aligned with international standards, particularly those outlined by Council of Europe (2025) European Directorate for the Quality of Medicines & Healthcare (EDQM) Guide to the quality and safety of organs for transplantation (9th ed.), ensuring adherence to evidence-based principles for donor selection, infectious risk assessment, and recipient safety^4^.

To address these challenges, a structured three-tier assessment model was implemented: an initial comprehensive donor evaluation at the donor hospital, including clinical assessment and first-line laboratory and serological screening; a second, organ-specific assessment to evaluate suitability and risks for each organ; and a third assessment at the organ recovery center, where a repeat set of laboratory and serological tests is performed to confirm safety prior to transplantation. In addition, detailed interviews with relatives are systematically incorporated into the risk assessment process to complement clinical and laboratory findings, particularly when historical medical information is limited [Council of Europe (2025) European Directorate for the Quality of Medicines & Healthcare (EDQM) Guide to the quality and safety of organs for transplantation (9th ed.)].^4^

### International collaboration as a fifth domain

Our experience in the UAE demonstrates that international collaboration is not merely supportive but essential for program growth. Partnerships with Spain, the United States, and regional Gulf states have provided training, expertise, and reciprocal organ sharing to enable sustainable growth. As outlined by Neuberger et al, non-utilization of organs due to various reasons from consented deceased donors remains a significant factor in limiting access to transplantation facing mature transplant programs worldwide [10]. International collaboration could help mitigate challenges posed by small population size, cultural diversity, and limited early infrastructure to support the growth of deceased organ donation and transplantation.

Collaborative organ sharing programs in Europe such as the FOEDUS-EOEO portal (European Organ Exchange Organization; www.foedus-eoeo.eu) established in 2015 have highlighted the impact of cross-border organ allocation in enabling optimal utilization of deceased donor organs, while meeting the need of recipients including those with time sensitive need for transplantation [11]. In the US, while organ utilization rates have improved significantly, concepts such as allocation out of sequence have been used to allocate organs outside the match to minimize organ discard rates and improve utilization [12]. Organ sharing between different jurisdictions and countries is even more important in low deceased donation rate settings to help build sustainable deceased donation and transplantation programs as it lowers organ discard rates and provides positive reinforcement for organ donation teams, while allowing transplant programs to grow and succeed over time. Therefore, as opposed to individual countries achieving national self-sufficiency, which is a great aspirational goal that supports progress, regional self-sufficiency in deceased organ donation is a more realistic goal.

In the Middle East & Gulf region, there is a scarcity of established programs in deceased organ donation with much lower donation rates compared to North America and Europe, primarily due to historical lack of infrastructure, personnel and awareness among healthcare professionals and public. This, coupled with the growing disease burden of liver disease, primarily driven by metabolic-dysfunction associated steatotic liver disease (MASLD), highlighted the potential for regional organ sharing agreements based on the principle of reciprocity supported by various international consensus recommendations to help meet demand while ongoing efforts to develop deceased organ donor programs continued [13, 14]. It is worth noting that our successful collaborative model with KSA since the inception of solid organ transplantation in the UAE has continued to ensure the optimal utilization of abdominal and thoracic organs procured from deceased donors in the UAE to save maximum number of patients with organ failure.

Historically most patients in need of liver transplant in Kuwait and other member nations of the GCC except KSA, traveled to other countries, with attendant financial implications and also presented an unmet need for those with time sensitive need for LT including patients with fulminant liver failure (FHF), severe acute on chronic liver failure and high model for end stage liver disease (MELD) scores [6, 15]. This presented a unique opportunity to establish a collaborative organ sharing arrangement to leverage the geographical proximity of the two countries and alignment among transplant leadership to provide timely access to recipients in both countries in need of liver transplant, while simultaneously optimizing utilization of deceased donor organs. A successful regional organ sharing arrangement was initiated with Kuwait in December 2021, that has since grown significantly (Figure 2) This collaboration entailed negotiating bilateral national arrangements to ensure compliance with regulatory and legal requirements and establishing operational workflows to ensure seamless cross-border organ transport that involved close coordination with immigration, law enforcement and diplomatic missions in both countries. Potential recipients in need of liver transplantation in the UAE and Kuwait have benefited from improved access to deceased donor organs as part of this collaborative organ sharing agreement.

**FIGURE 2:**
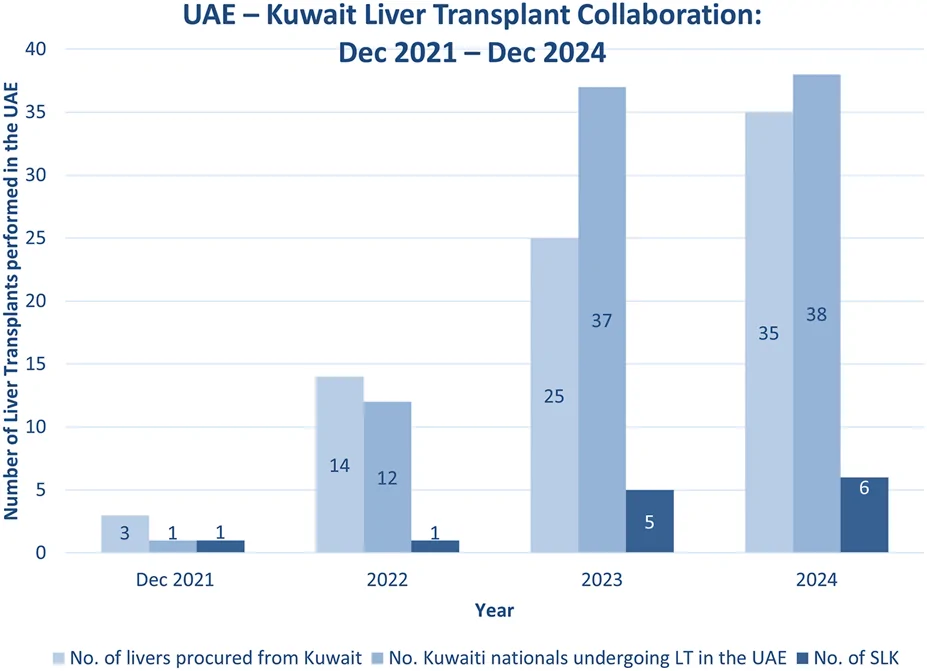
UAE–Kuwait liver transplant collaboration: December 2021 – December 2024. SLK, Simultaneous Liver Kidney transplant.

Solid organ transplantation was established in Kuwait based on the Ministerial decree in law No. 55 of 1987, followed by the formal establishment of a deceased organ donation program in 1996. Kuwait currently ranks 2nd in the Middle East Gulf region with 11.16 deceased donors PMP in 2024 (source: GODT data;^5^). The growth in deceased organ donation rates in the UAE and Kuwait reflects prior and ongoing initiatives related to voluntary organ donation. These efforts have placed a strong emphasis on strengthening the capacity of healthcare professionals through targeted training, education, and the development of standardized protocols. Moreover, by integrating deceased organ donation into the routine operations and policies of the healthcare system, these initiatives have normalized the practice, making it an expected and essential component of end-of-life care. This systemic approach has helped ensure that opportunities for organ donation are consistently identified and acted upon, thereby contributing to the sustained growth in donation rates. The ‘Declaration of Istanbul on Organ Trafficking and Transplant Tourism’, the goal of which was to foster safe and accountable practices that meet the needs of transplant recipients while protecting donors also supports equitable allocation of organs shared between jurisdictions and countries, prompting the UAE and Kuwait to start active organ sharing of non-utilized organs [13]. The population of the UAE is more than twice that of Kuwait, resulting in access to a significantly larger pool of deceased organ donors to critically ill recipients with liver failure in both countries. Diligent collaborative efforts between the liver transplant team at CCAD, NCDT, transplant leadership and hepatologists in Kuwait has ensured the seamless transition of patients between the two countries before and after transplantation. Kuwait’s national organ donation and transplant program has worked diligently over the years to increase public awareness of deceased organ donation. ‘Hayat’ is the national system in the UAE, established with the goal to enhance organ donation in accordance with international standards and practices. We hope that ‘Hayat’ could also serve as a model for other countries in the region and help sustain progress made in increasing awareness of deceased organ donation among both the public and healthcare professionals.

As described earlier, even though deceased organ donation and transplantation in Kuwait were established years prior to this collaboration, Kuwaiti recipients often traveled abroad for liver transplant while a few transplants were performed in the country in collaboration with King’s College London [6]. The long-term goal of our collaborative relationship is to not just ensure timely access to liver transplant for Kuwaiti nationals but also support the formal establishment of liver transplantation in Kuwait [6].

UAE partnership with Kuwait transplant leadership has ensured the seamless transition of care once liver recipients return to Kuwait after transplant. This model has since been extended to include thoracic organ transplantation as well and holds great promise to bridge the supply demand gap and meet the needs of patients in both countries. Our approach is aligned with the 2024 World Health Assembly resolution on organ donation (WHA77.4, June 2024) that highlighted the need to promote structured, ethical international cooperation to combat shortage of donor organs and unethical practices.^6^ Our successful regional collaboration in organ donation and transplantation with Kuwait exemplifies the resolution’s call to promote sustainable, international cooperation for the exchange of organs and transplant services based on the principles of reciprocity and solidarity, with the goal of improving access to transplantation for more patients in need while ensuring ethical practices. Lessons learnt from this successful collaborative partnership could also potentially be expanded to other nations in the region that lack established infrastructure to support deceased organ donation, thereby helping avoid deceased donor organ non utilization.

## Results

Diligent efforts to integrate deceased organ donation into the UAE healthcare system has resulted in the exponential growth of deceased organ donation in the country, propelling the growth in liver transplant volumes as well. Figures 3, 4 highlight the growth in deceased organ donors in the UAE from inception, ranked as the nation with the fastest growth over the past 6 years, reaching 11.56 donors PMP in 2024. (Figure 3 source: Global Observatory on Donation and Transplantation (GODT) data, produced by the WHO- ONT collaboration).^5^ Over the course of the study period, even though the population of the UAE grew 13% (from 9.3 to 10.5 million), rate of deceased organ donation increased more than ten-fold from 0.8 PMP in 2018 to 11.56 in 2024 (Figure 4) highlighting our collective efforts in not only increasing awareness of deceased organ donation but also establishing the infrastructure to improve identification of potential brain-dead deceased donors.

**FIGURE 3:**
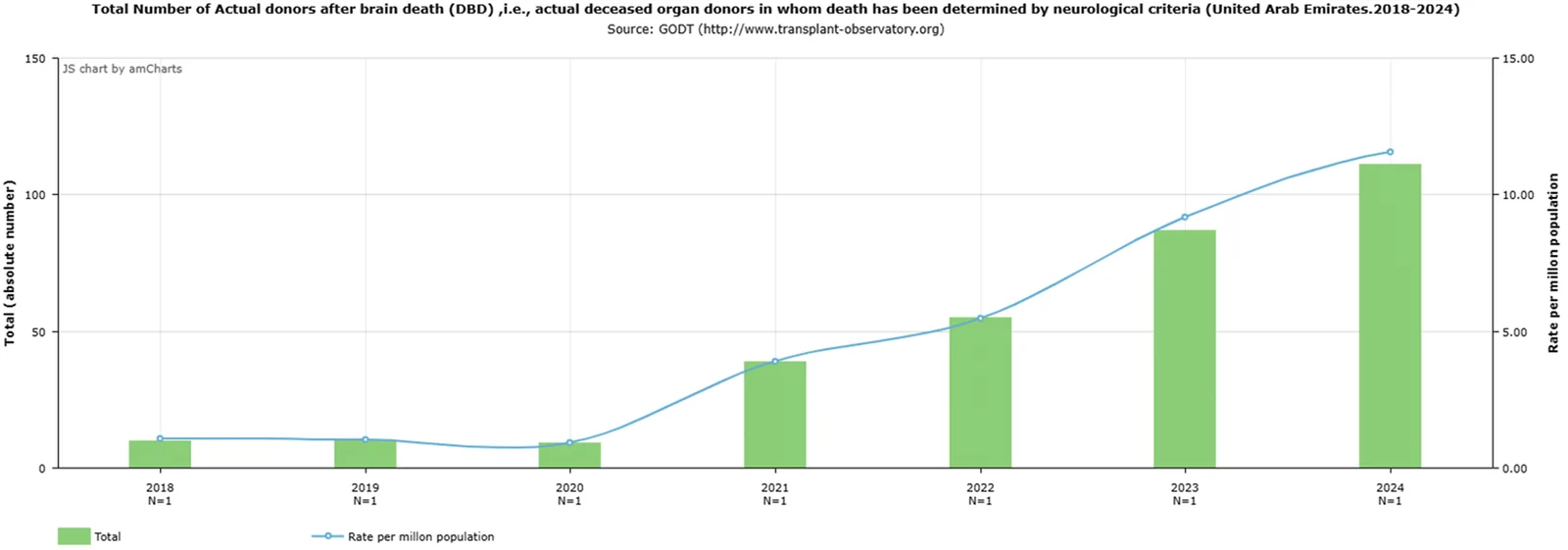
Total number of deceased organ donors in the UAE: 2018 -2024. Data are based on the Global Observatory on Donation and Transplantation (GODT) data, produced by the WHO- ONT collaboration https://www.transplant-observatory.org/data-charts-and-tables/chart/.

**FIGURE 4:**
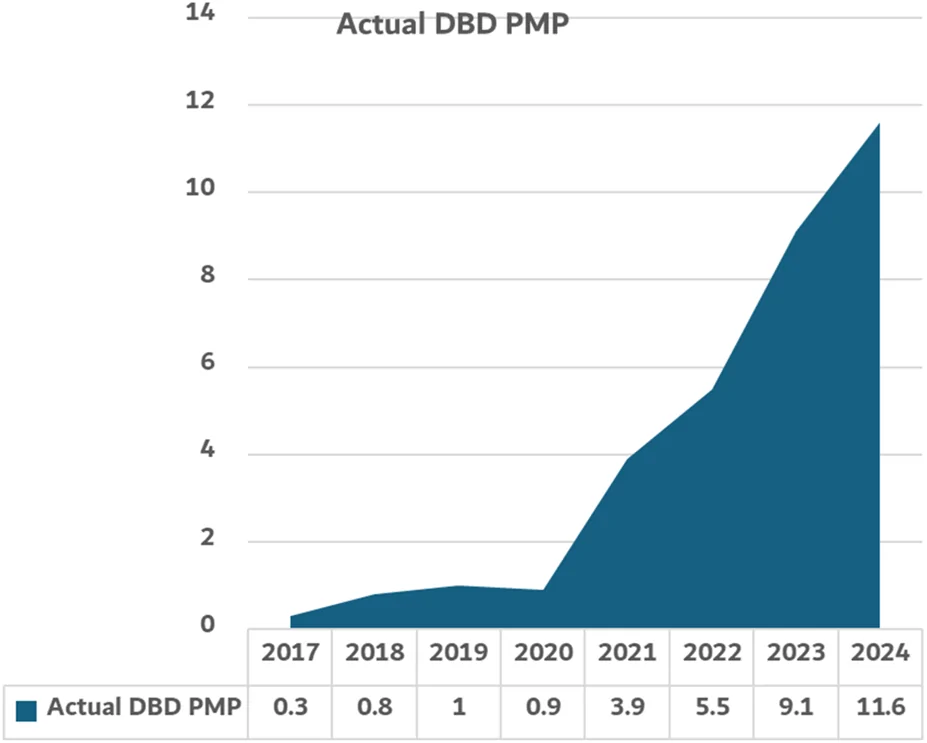
Donation after brain death in the UAE per million population: 2017–2024.

Also noteworthy is the fact that the number of organs transplanted per deceased donor in the UAE was 3.2 in 2023. This is likely due to several factors including the relatively young age of deceased donors, optimal donor management practices and, as highlighted in the next section, organ sharing collaboration with other countries in the region to avoid discarding viable organs not utilized by UAE transplant programs, which also served to respect the wishes of donor families wishing to impact as many lives as possible through the heroic act of organ donation. Residents of the UAE represent more than 200 nationalities, and the ‘Hayat’ program reflects this diversity and progress in deceased organ donation, with more than 53 nationalities either donating (Median age: 43.7; 77% male) or receiving organ transplants, benefitting liver recipients from 34 different nationalities (source: NCDT). While we await implementation of legislative initiatives aimed at expanding health insurance coverage for transplantation, a significant proportion of residents have insurance coverage for transplant with several charitable organizations in the UAE providing financial coverage for transplant for those in need. Moreover, critically ill recipients in the UAE with fulminant hepatic failure or severe acute on chronic liver failure are considered for transplant regardless of healthcare insurance coverage and recent legislative initiatives mandating coverage of transplantation by all healthcare insurance plans will help ensure universal access to organ transplantation to all UAE residents.

### Impact of coronavirus pandemic

The COVID-19 pandemic had a measurable impact on both organ donation and transplantation processes during the study period. Restrictions on movement between emirates affected the coordination and logistics of donor management, organ retrieval, and allocation. Despite these constraints, the system adapted by reorganizing transport pathways and strengthening local coordination, ensuring continuity of donation activities. Organ allocation processes were maintained, although operational timelines and coordination mechanisms required greater flexibility due to mobility limitations and evolving infection control requirements.

Clinically, the pandemic necessitated significant adjustments in protocols, including the safe utilization of organs from COVID-19–positive donors and, in selected cases, transplantation into COVID-19–positive recipients. Laboratory testing algorithms were adapted to incorporate SARS-CoV-2 screening, and infection prevention measures were reinforced across ICU and transplant settings. ICU capacity pressures and strict infection control protocols influenced donor identification and management, while cross-border transport limitations further challenged logistics. Nevertheless, these adaptations allowed the program to sustain transplantation activity under unprecedented conditions.

### Liver transplantation in the UAE: 2018–2024

Since the inception of LT in Feb 2018, 376 liver transplants were performed in the UAE until end of 2024. At CCAD, as of December 31, 2024, we performed 337 liver transplants – 303 from deceased donors and 34 LDLTs–including 27 simultaneous liver-kidney transplants, one combined liver-lung transplant and one patient who underwent combined coronary artery bypass grafting and deceased donor LT [16, 17]. Notably, among 19 recipients who underwent LT for FHF, 4 were emergent LDLTs prior to the growth in deceased organ donation. As is the case in the US, MASLD remains the primary driver of the need for LT in the region, with almost 15 percent of recipients having hepatocellular carcinoma. At CCAD, one year graft and patient survival rates were 93% (95% CI: 88.7–95.6) and 94.6% (95% CI: 90.7–96.9) respectively.

Recipients from the UAE and Kuwait are listed on a common waitlist maintained by the NCDT, with prioritization based on the MELD-based organ allocation policy followed by the United Network for Organ Sharing (UNOS) in the United States, as reported previously [2]. All LT as part of this collaborative initiative are performed at CCAD, the nation’s only multi-organ abdominal and thoracic transplant center. Since the inception of LT in Kuwait in 2018, the country had performed 11 DDLTs prior to the operationalization of this organ sharing arrangement in December 2021 [6]. Since then, until Dec 2024, we have successfully procured 77 livers and 10 kidneys from deceased organ donors in Kuwait and transplanted 88 patients from Kuwait, 13 of whom were recipients of simultaneous liver-kidney transplant (SLK), while 2 recipients from Kuwait underwent LDLT (Figure 2). Among patients from Kuwait transplanted at CCAD, 1 year liver allograft survival was 95.5% (95% CI: 83.2–98.8), with 1-year patient survival of 97.8% (95% CI: 85.1–99.7).

## Conclusions and future considerations

The successful growth of liver transplantation in the UAE was enabled by legal, regulatory, and financial support from several key stakeholders. However, several challenges and unmet needs would have to be addressed to enable its continued growth and success, given the anticipated exponential increase in disease burden related to MASLD that is expected to continue to drive the demand for liver transplant services in the Middle East [14].

Ongoing challenges include lack of coverage for transplant services in some of the insurance policies, need to improve public awareness of deceased organ donation and reforms in reimbursement models to reflect the complexity of liver transplantation. Several survey-based cross-sectional studies done in the region have highlighted the lack of public awareness regarding the concept of brain death and deceased organ donation [18]. Since solid organ transplantation from deceased donors was established relatively recently in the UAE, historically reimbursement for transplant services did not accurately reflect the care complexity involved and did not include costs associated with organ donation biovigilance logistics. These issues are being addressed now to ensure sustainability and continued growth of deceased donation.

We are hopeful that ongoing legislative and regulatory initiatives will enable us to reach our intended goal of improving organ donation rates and improved access to LT. Recent legislative initiatives include a federal mandate to include organ transplant coverage in all insurance policies and a draft proposal to legalize donation after circulatory death (DCD) LT. The former aims to mandate coverage for organ transplantation in all healthcare insurance policies, which should ensure universal access to organ transplant services for all UAE residents. DCD grafts have been shown to have comparable outcomes in patients with FHF and could help bridge the supply-demand gap in patients with FHF, severe acute on chronic liver failure and high MELD scores [19]. While expansion of regional organ sharing agreements could help meet the time sensitive need for transplant in these critically ill patients, we also hope to incorporate international scientific best practices in DCD transplantation to establish the region’s first program in DCD LT to further improve access. In this context, we have also incorporated machine perfusion in our transplant practice to enhance the quality and utilization of extended criteria liver grafts. Despite the significant growth in deceased organ donation, continued growth of LDLT also remains an ongoing priority. Other ongoing initiatives include continued expansion of organ donation units, transparent reporting of quality measures and outcomes and establishment of formal training programs in liver transplantation.

To summarize, 7 years after initiating liver transplantation, despite significant challenges, the UAE has made considerable progress in deceased organ donation and transplantation. Enhanced regional collaboration as exemplified by our collaboration with Kuwait, has helped meet the needs of patients in both countries, while also promoting organ donation awareness in a region where knowledge of which has historically been very limited and could potentially be expanded to other nations in the region that lack established infrastructure to support deceased organ donation. Our experience has shown that challenges unique to organ donation and transplantation could be overcome by collaboration among key stakeholders including regulatory, cultural, and religious authorities.

## Data Availability

The raw data supporting the conclusions of this article will be made available by the authors, without undue reservation.

## Footnotes

1
https://www.edqm.eu/en/-/newsletter-transplant-2021-now-available

2
https://u.ae/en/information-and-services/health-and-fitness/Blood-and-organ-donation/organ-donation-and-transplant

3
https://uaelegislation.gov.ae/en/legislations/2135/download

4 Council of Europe. (2025). European Directorate for the Quality of Medicines & Healthcare (EDQM). *Guide to the Quality and Safety of Organs for Transplantation* (9th ed.) Available online at: https://www.edqm.eu/en/guide-quality-and-safety-of-organs-for-transplantation.

5
https://www.transplant-observatory.org/data-charts-and-tables/chart/.

6
https://apps.who.int/gb/ebwha/pdf_files/WHA77/A77_R4-en.pdf

## References

[1] Kumar S Sankari BR Miller CM Obaidli AAKA Suri RM . Establishment of solid organ transplantation in the United Arab Emirates. Transplantation (2020) 104:659–63. 10.1097/TP.0000000000003030 32224809

[2] Kumar S Miller CM Hashimoto K Quintini C Kumar A Balci NC Liver transplantation in the United Arab Emirates from deceased and living donors: initial 2-Year experience. Transplantation (2021) 105:1881–3. 10.1097/TP.0000000000003455 34416746

[3] Johnston-Webber C Mah J Streit S Prionas A Wharton G Mossialos E A conceptual framework for evaluating national organ donation and transplantation programs. Transpl Int (2023) 36:11006. 10.3389/ti.2023.11006 37334013 PMC10273098

[4] Shaheen FA Al-Jondeby M Kurpad R Al-Khader AA . Social and cultural issues in organ transplantation in Islamic countries. Ann Transplant (2004) 9(1):11–14. 15478904

[5] Miller AC Ziad-Miller A Elamin EM . Brain death and Islam: the interface of religion, culture, history, law, and modern medicine. Chest (2014) 146:1092–101. 10.1378/chest.14-0130 25287999 PMC4188144

[6] Jamal M Almahmeed H Alghanem M AlMatooq M Sadek A AlMousawi M Organ transplantation in Kuwait and the recent initiation of a liver program. Transplantation (2021) 105:2125–7. 10.1097/TP.0000000000003537 34591813

[7] Masri M Haberal M . Solid-organ transplant activity in MESOT countries. Exp Clin Transplant (2013) 11(Suppl. 1):1–8. 10.6002/ect.2013.ecte3b 23577959

[8] Johnston-Webber C Mah J Prionas A Streit S Wharton G Forsythe J Solid organ donation and transplantation in the United Kingdom: good governance is key to success. Transpl Int (2023) 36:11012. 10.3389/ti.2023.11012 37305339 PMC10249499

[9] Greer DM Shemie SD Lewis A Torrance S Varelas P Goldenberg FD Determination of brain death/death by neurologic criteria: the world brain death project. JAMA - J Am Med Assoc (2020) 324(11):1078–97. 10.1001/jama.2020.11586 32761206

[10] Neuberger J Callaghan C . Organ utilization – the next hurdle in transplantation? Transpl Int (2020) 33(12):1597–1609. 10.1111/tri.13744 32935386

[11] Elmer A Lütolf VV Carella C Beyeler F Krügel N Straka L Importance and potential of European cross-border deceased donor organ allocation through FOEDUS-EOEO platform. Transpl Int (2023) 36:11327. 10.3389/ti.2023.11327 38020752 PMC10643207

[12] Kulkarni S Adebiyi O Bullock S Chen G Dillon J Jokimaki L An ethical analysis of allocating organs out of sequence. Curr Transpl Rep (2025) 12(1):29. 10.1007/s40472-025-00486-4

[13] Steering Committee of the Istanbul Summit. Organ trafficking and transplant tourism and commercialism: the declaration of istanbul. The Lancet (2008) 372(9632):5–6. 10.1016/S0140-6736(08)60967-8 18603141

[14] Razavi H Abaalkhail F Al Khathlan A Alghamdi M Al Hamoudi WK Nonalcoholic fatty liver disease burden – saudi Arabia and United Arab Emirates, 2017–2030. Saudi J Gastroenterol (2018) 24(4):211–9. 10.4103/sjg.sjg_122_18 29956688 PMC6080149

[15] L’Hermite S Coirier V Artru F . Liver transplantation in the context of acute-on-chronic liver failure (ACLF): where do we stand in 2025? Transpl Int (2025) 38:14752. 10.3389/ti.2025.14752 40837942 PMC12360965

[16] Kumar S Iuppa G Quintini C Al Obaidli A Gomez M Diago-Uso T V-113.3: growth and success of liver transplantation in the United Arab Emirates: an interim 6-Year report. Transplantation (2024) 108(9S):411. 10.1097/01.tp.0001066736.52395.d0

[17] Kumar S Al Obaidli A Al Mousawi M Gomez M Quintini C Iuppa G P.380: collaborative organ sharing in the Middle East: the United Arab Emirates - kuwait liver transplant experience. Transplantation (2024) 108(9S):639. 10.1097/01.tp.0001068276.14310.64

[18] Shaheen MF Alghafees MA Alzeer ZA Altheaby A Shaheen FAM . Barriers to deceased donor procurements: the Saudi experience. Transplantation (2023). 10.1097/TP.0000000000004507 37341713

[19] Kumar S Lin S Schold JD . Impact of donation after circulatory death donor allografts on outcomes following liver transplantation for fulminant hepatic failure in the United States. Am J Transplant (2020) 21(1):382–390. 10.1111/ajt.16286 32865321

